# Streptococcus agalactiae Meningitis in an Adult: A Case Report

**DOI:** 10.7759/cureus.76888

**Published:** 2025-01-03

**Authors:** Iftikhar Ahmad, Salma Abbas, Ali Anjum, Summiya Nizamuddin, Azra Parveen

**Affiliations:** 1 Department of Internal Medicine/Infectious Diseases, Shaukat Khanum Memorial Cancer Hospital and Research Centre, Lahore, PAK; 2 Department of Medicine, Shaukat Khanum Memorial Cancer Hospital and Research Centre, Lahore, PAK; 3 Department of Internal Medicine, Shaukat Khanum Memorial Cancer Hospital and Research Centre, Lahore, PAK; 4 Department of Microbiology, Shaukat Khanum Memorial Cancer Hospital and Research Centre, Lahore, PAK

**Keywords:** burkitt lymphoma, immunocompromised, infectious disease medicine, meningitis, streptococcus agalactiae

## Abstract

*Streptococcus agalactiae* meningitis is usually observed among neonates. We present a rare case of *Streptococcus agalactiae* meningitis in a 33-year-old immunocompromised male patient with Burkitt lymphoma. Initially, the patient exhibited nonspecific symptoms, including postprandial vomiting, blurred vision, and episodic memory loss. Cerebrospinal fluid (CSF) analysis and imaging revealed meningitis. Empiric antibiotic therapy with ceftriaxone and vancomycin, followed by targeted ampicillin treatment, resulted in complete resolution. This case highlights the importance of considering unusual pathogens in immunocompromised patients with nonspecific symptoms and underscores the need for prompt diagnosis and targeted antibiotic therapy.

## Introduction

Meningitis is characterized by inflammation of the meninges and tissue surrounding the brain and spinal cord. Prompt evaluation and management of meningitis is recommended as any delay may lead to increased mortality [1]. Several microorganisms including bacteria, fungi, and viruses are implicated in meningitis. The causative organisms for meningitis vary based on age. For adults, the most common pathogens are *Streptococcus pneumoniae *and *Neisseria meningitidis, *causing 75%-90% of cases, while in neonates, *Streptococcus agalactiae*, *Escherichia coli*, and *Listeria monocytogenes* account for the majority of cases [2]. Group B* Streptococcus *(GBS) is the most common cause of meningitis in neonates; however, it can rarely cause meningitis in adults. To the best of our knowledge, no such case has been reported in Pakistan. Here, we report a case of a young male patient with Burkitt lymphoma who was diagnosed with* Streptococcal agalactiae* meningitis.

## Case presentation

A 33-year-old male patient with Burkitt lymphoma underwent chemotherapy with dose-adjusted etoposide, prednisone, vincristine, cyclophosphamide, doxorubicin, and rituximab (DA-EPOCH-R) regimen until April 2024, followed by a scheduled in-patient admission in early May 2024 for continued treatment.

On May 6, 2024, the patient developed persistent postprandial vomiting, concurrent blurred vision, episodic memory loss, and decreased oral intake. Notably, he remained asymptomatic for epigastric pain, hematemesis, diarrhea, headache, and loss of consciousness.

**Figure 1:**
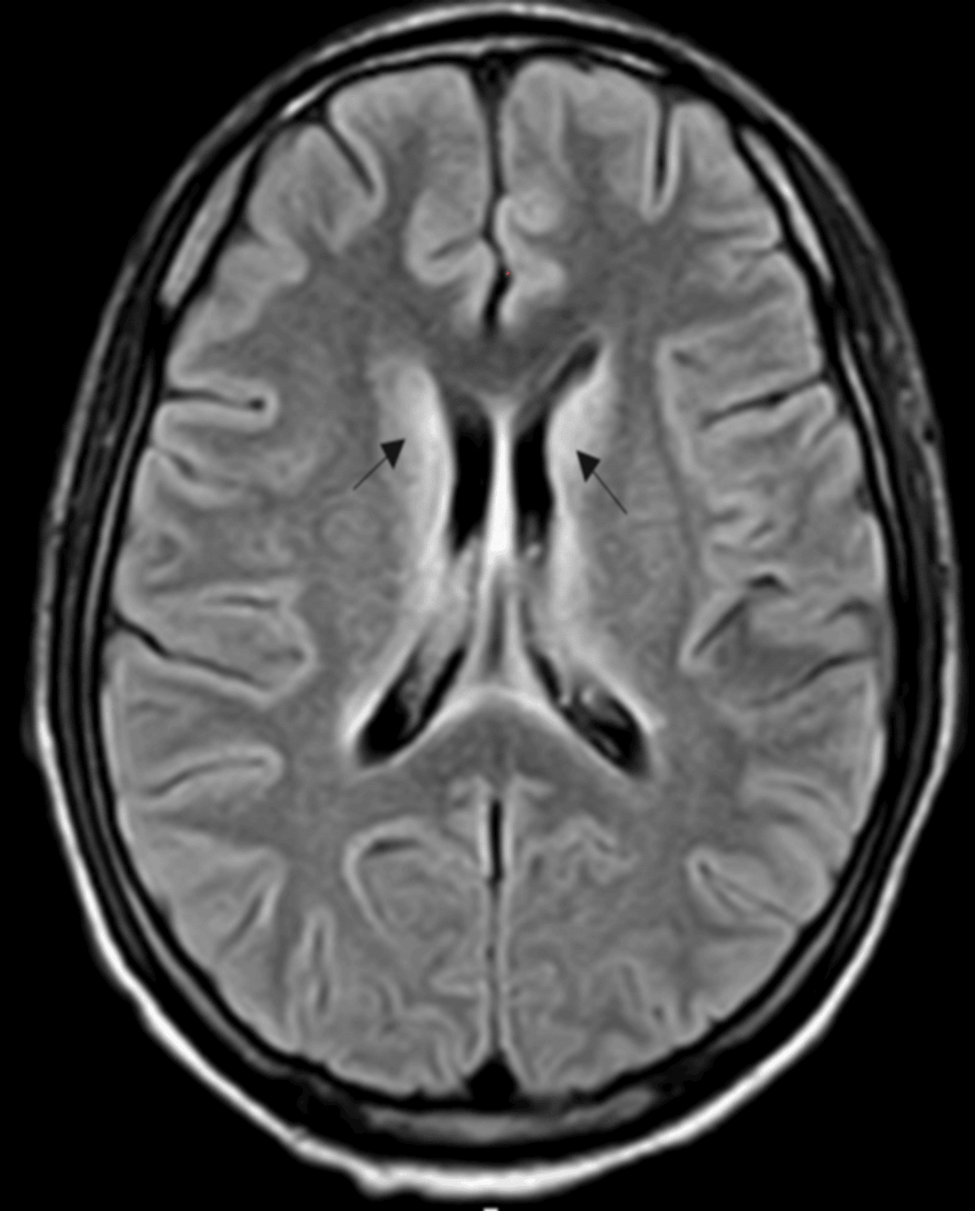
Brain MRI showing periventricular hyperintense signals (black arrows) MRI: magnetic resonance imaging

A brain magnetic resonance imaging (MRI) performed on the same day revealed bilateral symmetrical hyperintensities in the periventricular white matter, medial thalamus, periaqueductal grey matter, and crus cerebri on T2-weighted and fluid-attenuated inversion recovery (FLAIR) sequence (Figure 1).

The initial cerebrospinal fluid (CSF) analysis yielded inconclusive results (Table 1), intravenous thiamine was administered following consultation with the neurology team, suspecting Wernicke encephalopathy in view of brain MRI findings, and the patient was subsequently discharged.

**Table 1:** CSF analysis at first presentation CSF: cerebrospinal fluid

Parameters	Values	Normal range
White blood cells (mm^3^)	0	0-5
Red blood cells (mm^3^)	0	0
Glucose (mg/dL)	81	50-80
Protein (mg/dL)	34	15-45
Meningitis BioFire array	Negative	Negative

Nine days later, the patient revisited the emergency department with a two-day history of recurrent vomiting with a frequency of 5-6 episodes daily, vertigo, and visual disturbances. He denied experiencing fever, headache, chest pain, auditory symptoms, or limb weakness.

Upon evaluation, his vital signs were within normal limits, including blood pressure (110/75 mm Hg), pulse rate (84 bpm), oxygen saturation (96% on room air breathing), and axillary temperature (98.6°F).

Physical examination revealed bilateral diplopia on extreme horizontal gaze without ophthalmoplegia and signs of meningeal irritation. The remainder of the neurological and systemic examinations were unremarkable. Baseline investigations, encompassing complete blood picture, serum electrolytes, and renal and liver function tests, were within normal parameters (Table 2).

**Table 2:** Baseline investigations

Parameters		Values	Normal range
Complete blood picture	White blood cells (×10^3^/µL)	6.63	4-11
	Hemoglobin (g/dL)	11.4	13.2-16.7
	Platelets (×10^3^/µL)	187	150-450
Serum electrolytes	Sodium (mmol/L)	137	135-145
	Potassium (mmol/L)	4.2	3.5-5.1
	Chloride (mmol/L)	106	98-107
Renal function tests	Blood urea nitrogen (mg/dL)	15.16	9-22
	Creatinine (mg/dL)	0.9	0.90-1.30
Liver function tests	Total bilirubin (mg/dL)	0.8	0.3-1.0
	Alanine aminotransferase (U/L)	38	<45
	Aspartate aminotransferase (U/L)	27	<35
	Alkaline phosphatase (U/L)	156	56-167

Cardiac assessment, including echocardiography, was unremarkable.

**Figure 2:**
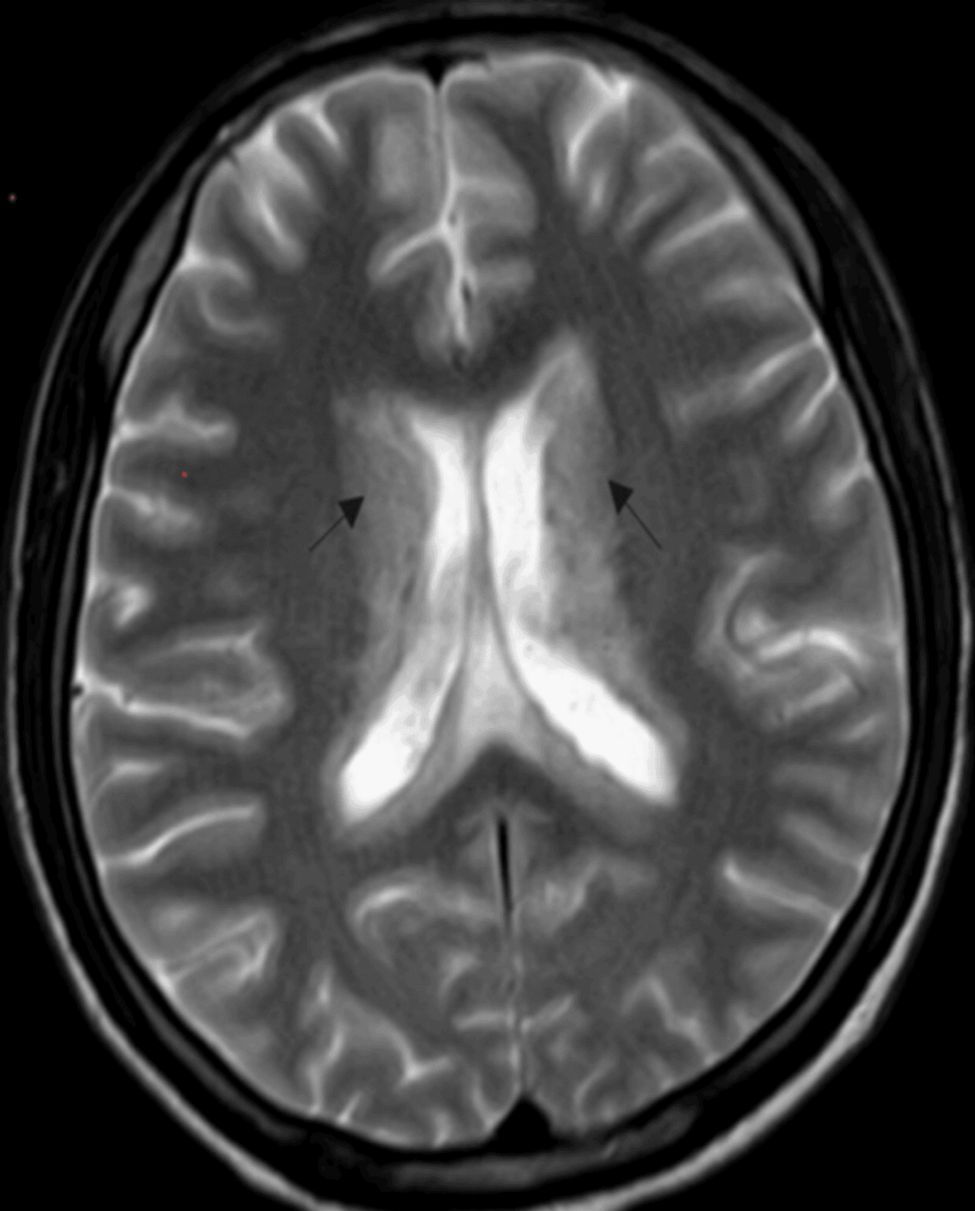
Brain MRI showing interval increase in periventricular hyperintense signals (black arrows) MRI: magnetic resonance imaging

Subsequent brain magnetic resonance imaging (MRI), on the day of his second in-hospital admission, demonstrated an interval increase in hyperintense signals on T2-weighted and fluid-attenuated inversion recovery (FLAIR) sequences (Figure 2).

Cerebrospinal fluid (CSF) analysis revealed an elevated white blood cell count with neutrophil predominance, elevated protein, and decreased glucose levels (Table 3).

**Table 3:** CSF analysis at second presentation CSF: cerebrospinal fluid

Parameters	Values	Normal range
White blood cells (mm^3^)	420	0-5
Lymphocytes (%)	20	90-100
Polymorphonuclear cells (%)	80	0-10
Glucose (mg/dL)	29 (less than two-thirds of blood glucose)	50-80
Protein (mg/dL)	161	15-45
CSF culture	Streptococcus agalactiae	No growth
Meningitis BioFire array	Streptococcus agalactiae	Negative

Empiric treatment with ceftriaxone (2 gm IV q12h daily) and vancomycin (60 mg/kg IV daily) was initiated, followed by targeted therapy with ampicillin (100 mg/kg IV q6h daily).

**Figure 3:**
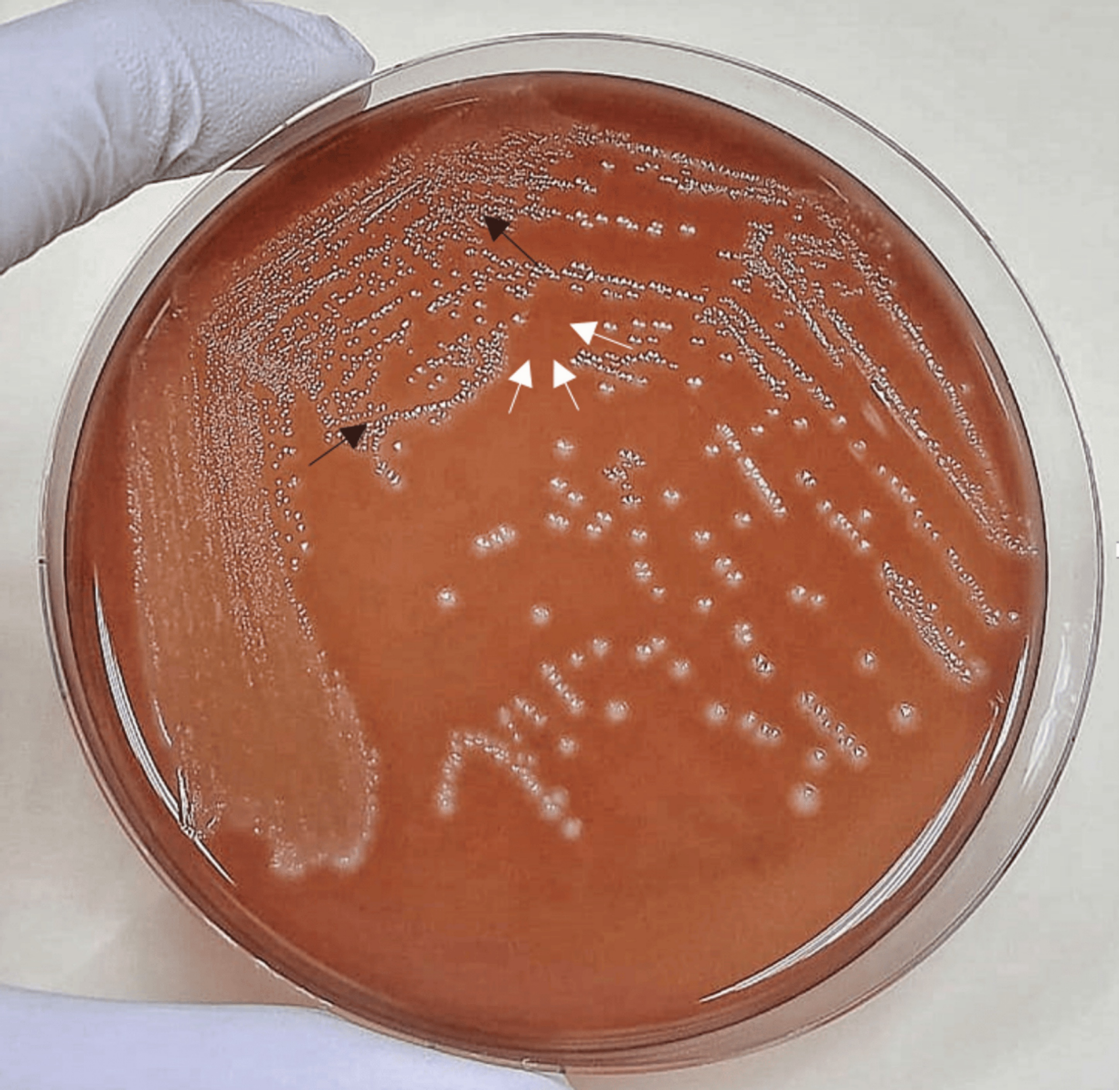
Grey-white colonies of Streptococcus agalactiae (black arrows) with surrounding beta hemolysis (white arrows) on blood agar

CSF culture on blood agar yielded grey-white colonies with a narrow zone of beta hemolysis (Figure 3), which were finally identified as gram-positive and catalase-negative cocci in chains. Meningitis BioFire film array panel was positive for *Streptococcus agalactiae*.

The patient completed a two-week duration of antibiotic, and his clinical status improved significantly, with subsequent CSF analysis showing complete resolution. He was discharged home and scheduled for further chemotherapy. A follow-up visit two weeks later revealed no active complaints.

## Discussion

*Streptococcus agalactiae*, a group B *Streptococcus* (GBS) according to the Lancefield classification, is a gram-positive, chain-forming facultative anaerobe. To date, 10 serotypes (1a, 1b, II-IX) of this bacterium have been identified based on the differences in its antigens on polysaccharide capsules. GBS is considered a normal colonizer of gastrointestinal tracts and vagina. Although about 20%-30% of healthy adults are asymptomatic carriers of this microorganism, this seemingly benign bacterium is a major cause of meningitis and septicemia in neonates [3,4].

Moreover, in adults,* *GBS is associated with several invasive diseases, including skin, soft tissue, or bone infection (36%); bacteremia with no identified source (30%); urosepsis (14%); pneumonia (9%); and peritonitis (7%) [5]. Although *Streptococcus agalactiae* is a major cause of meningitis in neonates, in adults, GBS meningitis is rarely a clinical manifestation and accounts for less than 1% of cases of bacterial meningitis [6]. However, the rate of invasive group B* Streptococcus* infection including meningitis is on the rise [7,8].

Several risk factors have been identified for the development of GBS meningitis, which includes old age, diabetes mellitus, malignancy, alcoholism, renal failure, and liver cirrhosis [9]. Additionally, there are several cases reported in healthy individuals without any of the abovementioned conditions [10]. However, the diagnosis of* Streptococcus agalactiae* in immunocompetent individuals should prompt further evaluation, particularly for CSF leakage, bacteremia, and endocarditis [11].

Most cases of GBS meningitis cannot be distinguished on clinical grounds from meningitis caused by other microorganisms because the presentation is mostly similar, but it has been reported that GBS meningitis cases tend to present early owing to early encephalopathic features [12,13]. In comparison to other causes of meningitis, case fatality of GBS meningitis has been reported to be higher (34.4%), and serotype V is associated with higher mortality [8,14].

In our case, the patient was suffering from hematological malignancy and was on active chemotherapy (DA-EPOCH-R regimen), putting him at high risk for contracting GBS-associated meningitis. Our patient recovered fully without any neurological sequelae. To the best of our knowledge, this is the first case reported in Pakistan, emphasizing the need for awareness among clinicians in this region regarding GBS meningitis, especially in patients with the aforementioned risk factors.

## Conclusions

This case report presents a rare and intriguing instance of *Streptococcus agalactiae* meningitis in an adult patient with Burkitt lymphoma, highlighting an uncommon presentation of this infection. *Streptococcus agalactiae* is a well-established cause of neonatal meningitis, with adults rarely affected. Our case illustrates that immunocompromised individuals, particularly those with hematological malignancies, may present with atypical presentations of GBS infection, including meningitis.

This case expands the literature on *Streptococcus agalactiae* meningitis in adults. Prompt diagnosis and treatment is the key to improving clinical outcomes.
